# Retrospective analysis of post-mortem findings in domestic ducks and geese from non-commercial flocks in Sweden, 2011–2020

**DOI:** 10.1186/s13028-021-00614-x

**Published:** 2021-11-24

**Authors:** Désirée Seger Jansson, Faruk Otman, Elisabeth Bagge, Ylva Lindgren, Pernille Engelsen Etterlin, Helena Eriksson

**Affiliations:** 1 Department of Animal Health and Antimicrobial Strategies, National Veterinary Institute, 751 89 Uppsala, Sweden; 2 Department of Clinical Sciences, Faculty of Veterinary Medicine and Animal Science, Swedish University of Agricultural Sciences, Box 7054, 750 07 Uppsala, Sweden; 3 Department of Pathology and Wildlife Diseases, National Veterinary Institute, 751 89 Uppsala, Sweden

**Keywords:** Diagnostics, Domestic duck, Domestic goose, Hybrid duck, Mortality, Muscovy duck, Pathology

## Abstract

**Background:**

Small poultry flock ownership has become a popular hobby in Europe and North America in recent years but there is a general lack of information regarding bird health and welfare. This retrospective analysis of routine post-mortem cases of non-commercial anseriform poultry aimed at providing information on causes of mortality mostly in relation to mortality events. For this purpose, birds that were submitted for routine post-mortem diagnostics to the National Veterinary Institute (SVA) in Sweden in 2011–2020 were retrospectively reviewed to determine main causes of mortality.

**Results:**

Records from 79 necropsy submissions involving 120 birds (domestic ducks *n* = 41, Muscovy ducks *n* = 45, hybrid ducks *n* = 2 and domestic geese *n* = 32) were retrieved and analysed. Most submissions (72.2%) represented flock disease events and unexpected mortality was the most common cause of submission (70.9% of submissions). Twenty-two submissions (27.8%) were referred by veterinarians. There was a wide range of diagnoses of infectious and noninfectious aetiologies. Infectious causes of mortality included parasitic (19.2%), bacterial (13.3%), fungal (10.0%) and viral infections (3.3%) (at bird level of all 120 birds). Some of these infections such as duck virus enteritis (DVE), highly pathogenic influenza (HPAI H5N8) in Muscovy ducks and leucocytozoonosis (*Leucocytozoon* sp.) in all three species were most likely acquired from contact with wild free-living waterfowl. Generalised yeast infection (Muscovy duck disease) was diagnosed in Muscovy ducks and in a Muscovy duck/domestic duck hybrid. Other diseases were related to generalised noninfectious causes (27.5% of all birds) including diseases such as kidney disease, amyloidosis, cardiac dilatation, reproductive diseases and idiopathic inflammatory conditions. Nutritional or management-related diseases were diagnosed in 14.2% of all birds including rickets and gastrointestinal impaction/obstruction. Congenital/developmental, neoplastic, toxic and traumatic causes of mortality were rare.

**Conclusions:**

The information obtained in this study can be used to identify and evaluate risks and help owners and veterinarians to prevent disease and provide adequate veterinary care for non-commercial anseriform poultry.

## Background

All domesticated ducks originate from wild free-living mallards (*Anas platyrhynchos*) except Muscovy ducks, which is a separate species (*Cairina moschata*). Most domestic geese are derived from the graylag goose (*Anser anser*), while the Chinese and African goose are domesticated forms of the swan goose (*A*. *cygnoides)*. Today, ducks and geese are raised commercially in Asia, Europe and North America and some other areas for meat, eggs and foie gras production [1]. Commercial duck and goose meat production represents a minor poultry sector in Sweden, with 12,000–13,000 birds each slaughtered commercially in 2020 (pers. comm. Ingrid Medin, Swedish Food Agency, January 14, 2021).

In recent years, small non-commercial poultry flock ownership has gained widespread popularity in Europe and North America [2–5] and indications suggest that this is also true for Sweden. These birds are kept alongside the commercial population, often in mixed gallinaceous/anseriform flocks with access to water sources. The birds are kept for self-sufficient egg and meat production, companionship, breeding, exhibition in public collections, shows, and for conservation purposes. A small population of Swedish heritage breed ducks, Muscovy ducks and geese are preserved through non-profit conservation efforts. Additionally, there are domestic anseriform birds of purebred, unknown or mixed genetic background. The population size and locations of flocks in Sweden are unknown.

Published international information on commonly occurring diseases in non-commercial anseriform poultry is scarce and research on diseases of commercial anseriform populations is focused primarily on infectious diseases, mostly of viral origin, and may not necessarily be representative for small flocks. Hence, there are valid motives for gaining better insight into the health and welfare of con-commercial poultry of all species, including ducks and geese. The number of non-commercial poultry submitted for routine necropsy to the National Veterinary Institute (SVA) has increased substantially in recent years. However, anseriform birds still represent a small number. A large majority of post-mortem examinations of poultry in Sweden are performed at SVA and can thus be considered as representative for the country. The aim of this single-centre study was to provide comprehensive information on causes of mortality in small flocks of anseriform poultry through retrospective analysis of routine necropsy submissions in 2011–2020.

## Methods

### Case selection, data retrieval and interpretation

Case accession reports of anseriform poultry, i.e., domesticated birds derived from the wild mallard, Muscovy duck, greylag goose and swan goose, originating from non-commercial (hobby) flocks that were submitted for diagnostic post-mortem examination to SVA between January 1, 2011, and December 31, 2020, were retrospectively analysed. Submissions from commercial farms, game birds (non-domesticated mallards raised for release in nature) and ornamental waterfowl (wild species of ducks, geese and swans kept in captivity) were not included. The same applied to submissions consisting of formalin-fixed biopsy specimens alone from any kind of anseriform bird. Submitter information was retrieved to include geographic location (county), owner category (private, 4**-**H youth organisation farm, heritage centre/public collection, natural resources/agricultural school), month of arrival, species, breed, number of submitted carcasses, age, means of death (dead/euthanised), case history (such as mortality, lameness, diarrhoea) and whether there was a referring veterinarian. Birds were considered as juveniles until eight weeks of age when the first adult moult usually starts, as adults from one year of age and as subadults when they fell between these age categories. Flock information such as flock size, other species, bird origin, vaccination status and medication were rarely available and were therefore not included. Records of parasites, gross and microscopic findings, results of ancillary diagnostic tests and diagnoses as noted by attending case coordinators were reviewed and assessed by an experienced poultry pathologist. In some cases, additional slides for histopathology, re-stains or additional stains were prepared and examined. Findings that were considered to be related to the same disease process were grouped together as one diagnosis and one or several diagnoses that were considered to contribute to disease/mortality were entered for each bird. Incidental independent findings were listed and not limited in number. When submissions included several birds, test results made from pooled samples or a subset of birds were entered for all carcasses in the same submission if similar gross or microscopic findings were observed. Diagnoses were categorized based on a previously published paper [6] as belonging to different categories: viral, bacterial, fungal, parasitic, neoplastic, generalised noninfectious, nutritional/management-related, traumatic, toxic, congenital/developmental and undetermined. Results were compiled at individual bird level as frequency (*n*) and per cent (%).

During the study period, owners of non-commercial poultry in Sweden were entitled to reduced cost post-mortem examinations. Co-funding was provided by the Swedish Board of Agriculture through the advisory company Farm & Animal Health (https://www.gardochdjurhalsan.se/) and the purpose was to gain insight into the animal health situation of non-commercial poultry. Co-funding was granted if there was a flock health problem i.e., a minimum of three diseased or dead birds during the month preceding post-mortem examination. Necropsies, microscopy and, to some extent, ancillary tests were covered if the pathologist deemed it necessary in order to achieve a diagnosis. During the study period (2011–2020) post-mortem examinations were provided free of charge until 31 December 2013 after which the costs gradually increased. Field veterinary consultation and services, shipping/delivery and post-mortem examinations of non-flock-related mortality were covered by owners. This also applied to some ancillary tests and molecular diagnostics that were covered by owners unless declined, with the exception of molecular diagnostics for avian influenza (avian influenza virus, AIV) and Newcastle disease (avian paramyxovirus type 1, APMV-1). No routine surveillance activities for viral diseases, zoonotic microorganisms or heavy metals involving non-commercial poultry were conducted in Sweden during the study period. Carcasses were shipped to the laboratory by delivery service or by car.

### Pathology and ancillary tests

Post-mortem examinations were performed by poultry pathologists according to a routine in-house poultry necropsy protocol (SVA). Birds were examined for presence of external and gastrointestinal parasites by pathologists. Body weight and sex were recorded. Samples for histopathology were collected at the discretion of the pathologist, fixed in 10% neutral buffered formalin, routinely processed, embedded in paraffin wax and cut at 4 µm. Sections were stained with haematoxylin & eosin (HE) and additional stains were performed when necessary, including Gram, periodic acid-Schiff reaction (PAS), Grocott’s methenamine silver, Ziehl–Neelsen acid fast, Pearl’s iron and Congo red stains.

When bacterial infection was suspected, samples were collected from one or several birds per submission after surface searing. Bacterial culture was conducted on the same day aerobically at 37 °C on 5% equine blood agar and bromcresol lactose purple agar plates. Anaerobic cultures were made on fastidious anaerobe agar plates (FAA, Lab M) at 37 °C. Plates were assessed at 24 and 48 h of incubation. When *Erysipelothrix* sp. infection was suspected, samples were pooled as per organ and pre-enriched in a selective sodium-azide crystal-violet broth (SVA) before routine culture. For *Listeria* sp. suspected samples were pooled and pre-enriched in enrichment broth for *Listeria* (Oxoid) with acriflavine hydrochloride, nalidixic acid and cycloheximide and incubated at 30 °C for 48 h followed by culture on Oxford agar (Oxoid) supplemented with *Listeria* selective supplement (Oxoid, SR0206), at 37 °C and routine culture as previously described. Bacterial species confirmation was done by colonial morphology, biochemical tests and/or MALDI-TOF MS technique (Bruker, Germany). Samples for *Salmonella* were cultured according to reference method ISO 6579–1:2017 [7]. Attempts to isolate yeasts were made on Sabouraud dextrose agar (SVA) at 30 °C and plates were read after 5–7 d.

Samples for detection and characterization of APMV-1 and AIV were collected from all birds in suspected submissions, pooled per organ and analysed by real-time qPCR based on European Union Reference Laboratory (EURL) recommended and additional published protocols [8–11]. PCR for duck enteritis virus (DEV) and goose parvovirus/Muscovy duck parvovirus (PGV/MDPV) were performed by Ceva-Phylaxia Co. Ltd., Ceva Animal Health, Budapest, Hungary as described [12–14]. For diagnostic confirmation of leucocytozoonosis and disseminated yeast infection, samples from lung, liver and spleen from a subset of submissions were analysed by PCR. Templates were prepared from frozen tissue by the Qiagen DNeasy Blood and Tissue Kit® (spin column protocol for purification of total DNA from animal tissues), Qiagen AB, Solna, Sweden) according to the manufacturer’s instructions and used at 1:1 and 1:10 dilution. For avian malaria (*Leucocytozoon* spp., *Haemoproteus* sp./*Plasmodium* sp.) conventional duplex non-nested PCR targeting the cytochrome b genes based on primers and cycling conditions as previously described [15] were applied. A case from a duck with hepatic megaloschizonts was used as positive control for *Leucocytozoon* spp. For yeasts, several primer combinations (ITS1, ITS4, NS5, NS8, NL-1 and NL-4) targeting fungal partial 18S and 26S rRNA genes were used [16, 17]. Ultra-pure water and tissues from a duck with avian pasteurellosis served as negative controls. PCR products were visualized on 1.5% agarose gels with SYBR® Safe DNA gel stain (Invitrogen, Eugene, OR, USA). Sequence reactions to identify the yeast were performed with the BigDye® Terminator v.3.1 kit (Applied Biosystems) in an ABI PRISM® 2700 Genetic Analyzer at Uppsala Genome Center (UGC), Uppsala University, Uppsala, Sweden. The nucleotide sequences were edited using the software package CLC Sequence Work Bench software, version 6.4 (CLC bio, Århus N, Denmark). A search for related sequences was made by the nucleotide-nucleotide BLAST application of the GenBank database.

## Results

### Submissions and birds

**Fig. 1:**
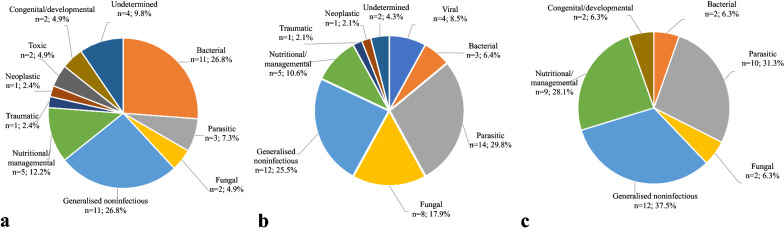
Diagnostic categories (%) contributing to mortality in non-commercial anseriform birds as determined at post-mortem examination. Birds were examined at the National Veterinary Institute (SVA) in Sweden in 2011–2020; **a** domestic duck (*n* = 41), **b** Muscovy duck (*n* = 45) including Muscovy duck/domestic duck hybrids (*n* = 2) and **c** domestic goose (*n* = 32). Percentages may exceed 100% because some birds were assigned multiple diagnoses

Seventy-nine necropsy submissions (1–5 birds each) involving a total of 120 domestic anseriform birds from 2011–2020 were reviewed (Table 1). Eight submissions (10.1%) involved more than one poultry species (one duck/goose and seven anseriform/chicken submissions). Most submissions originated from privately owned flocks (*n* = 63, 79.9%), and the remaining cases were received from heritage centres/public collections (*n* = 11, 13.9%), 4-H youth organisation farms (*n* = 3, 3.8%) or natural resources/agricultural schools (*n* = 2, 2.5%). Twenty-two submissions (27.8%) were referred by veterinarians. Birds were submitted all year around with a distinct peak during summer months (57.5% arrived in June–August). Birds were submitted from 18 out of 21 counties, including the southernmost and northernmost ones. The following duck breeds were submitted: heritage breeds (Yellow Swedish duck or Blue Swedish duck, 10 submissions, 17 birds), Pekin duck (3 submissions, 5 birds), Indian runner duck (1 submission, 2 birds) and Black East Indian duck (1 submission, 1 bird). Information on breed was missing in 14 submissions representing 17 birds. Goose submissions included heritage breeds (Skåne goose: 11 submissions, 12 birds; Öland goose, 4 submissions, 7 birds), and others including Chinese goose (1 submission, 2 birds), American Buff (1 submission, 1 bird) and unknown/mixed breed (8 submissions, 10 birds). Information on breed was not provided by owners of Muscovy ducks and there were two Muscovy duck/domestic duck hybrids. Information on age categories, sex and body condition are shown in Table 1. Bird age ranged from to 1 week to 8 years. In total, 57 submissions involved flock disease (domestic ducks 81.5%, Muscovy ducks and hybrid ducks 85.2%, domestic goose 48.0%). The most common cause of submission was increased mortality (56 submissions, 70.9%), often in combination with non-specific signs of weakness, listlessness, inappetence, respiratory distress, or diarrhoea. One case involved suspected rodenticide poisoning. Other causes of submission were weakness/listlessness or loss of condition (*n* = 10, 12.7%), locomotory problems (*n* = 5, 6.3%), respiratory signs (*n* = 4, 5.1%), signs related to the central nervous system (*n* = 2, 2.5%), skin conditions (*n* = 1, 1.3%) and diarrhoea (*n* = 1, 1.3%) in absence of mortality. Ninety-nine birds (82.5%) were found dead and 21 (17.5%) had been euthanised. Histopathology was performed in 68 out of 79 submissions (86.0%). A summary of ancillary tests performed to support diagnostics is shown in Table 2. Diagnostic categories are presented in Fig. 1.

**Table 1:** Sex, age categories and body condition in non-commercial anseriform poultry submitted for post-mortem diagnostics to the National Veterinary Institute (SVA), Sweden in 2011–2020

	Domestic duck	Muscovy duck	Hybrid duck	Domestic goose	Total	%
*N* (submissions^a^)	27	25	2	25	79	
*N* (birds)	41	45	2	32	120	
Sex						
Male	13	18	0	9	40	33.3
Female	25	21	2	23	71	59.2
No record	3	6	0	0	9	7.5
Age category						
Juvenile (0– < 8 weeks)	11	18	1	12	42	35.0
Subadult (8 weeks–˂1 year)	13	17	0	7	37	30.8
Adult (≥ 1 year)	17	10	1	13	41	34.2
Body condition						
Emaciated	1	6	0	8	15	12.5
Below normal	20	20	2	14	56	46.7
Normal	19	19	0	7	45	37.5
Above normal/obese	0	0	0	3	3	2.5
No record	1	0	0	0	1	0.8

^a^Each submission involved 1–5 birds

**Table 2:** Ancillary diagnostic tests performed to support post-mortem diagnostics of non-commercial anseriform poultry at the National Veterinary Institute (SVA), Sweden in 2011–2020

Diagnostic test	Domestic duck		Muscovy duck^a^		Domestic goose		Total	
	Subm	*N*	Subm	*N*	Subm	*N*	Subm	*N*
Bacterial culture, aerobic	7	11	9	13	6	10	22	34
Bacterial culture, enrichment^b^	0	0	1	2	1	1	2	3
Bacterial culture, *Salmonella*	2	2	2	2	3	3	7	7
Bacterial culture, anaerobic	0	0	1	1	0	0	1	1
Fungal culture (yeast)	0	0	1	2	0	0	1	1
PCR, avian influenza virus (AIV)	1	6	2	10	0	0	3	16
PCR, avian paramyxovirus-1 (APMV-1)	1	6	2	10	0	0	3	16
PCR, duck enteritis virus (DEV)	0	0	3	13	0	0	3	13
PCR, goose/Muscovy duck parvovirus (GPV/MDPV)	0	0	1	1	0	0	1	1
PCR, avian malaria^c^	1	1	3	9	0	0	4	10
PCR, 18S rDNA (fungal primers)	0	0	3	9	0	0	3	9
Virus isolation	0	0	2	9	0	0	2	9

*subm*  submission

^a^Including two Muscovy duck/domestic duck F1 hybrids

^b^Enrichment culture for *Erysipelothrix* sp./*Listeria* sp.

^c^*Leucocytozoon* sp., *Haemoproteus* sp./*Plasmodium* sp.

### Viral diseases

**Fig. 2:**
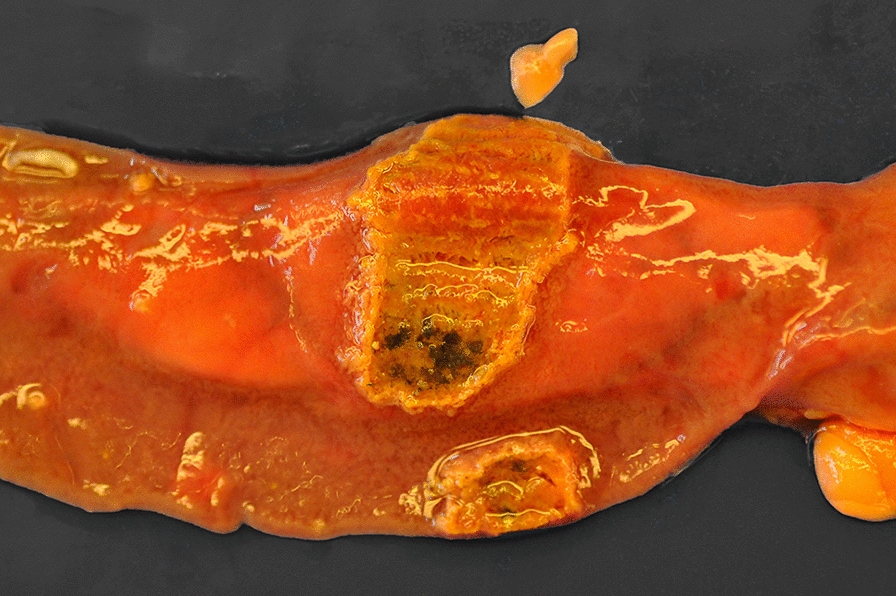
Annular necrotic band in jejunum of a Muscovy duck diagnosed with duck virus enteritis (DVE)

Viral diseases were diagnosed in three submissions (3.8%) involving four (3.3%) adult Muscovy ducks. Duck virus enteritis (DVE) caused by DEV/Anatid herpesvirus-1 was confirmed by PCR in two unrelated submissions in spring/summer of 2014 and 2015, respectively. In one of the flocks, mortality reached 80% during the two weeks preceding submission, and birds were weak. In the other flock, two birds were found dead in a pond. Affected birds were adults in normal body condition. Gross lesions included petechial to diffuse haemorrhage in a variety of organs, haemorrhagic intestinal contents, dark, mottled and enlarged spleen, white multifocal pinpoint foci in liver, multifocal to diffuse fibrinous pseudomembranes and pale annular bands in jejunum, colon and cloacal mucosae that were apparent from mucosal and serosal aspects (Fig. 2). Microscopically, intravascular thrombi, widespread haemorrhage and lymphocytolysis, necroses in liver, spleen and intestines and eosinophilic intranuclear inclusion bodies were observed.

Highly pathogenic avian influenza subtype H5N8 was diagnosed in February 2017 in an 8-months-old Muscovy duck belonging to a mixed-species poultry flock in southern Sweden. The cause of submission was 50% mortality in Muscovy ducks and chickens overnight. At necropsy, the duck was in normal condition and the ovary was active. Numerous petechial to ecchymotic haemorrhages were observed around the laryngeal opening, on the epi- and endocardium, in the liver, proventricular mucosa, small intestine, mesentery, thigh musculature and in body fat. The spleen was enlarged and dark. Profuse haemorrhage was observed in spleen and ovarian follicles. Unclotted blood was present in the coelomic cavity, and intestinal contents were blood tinged. Egg yolk was present on serosal linings. Main microscopic findings included severe congestion, widespread vascular thrombosis and haemorrhage, splenic lymphocytolysis, multifocal hepatocellular and myocardial necroses and perivascular mononuclear inflammation and gliosis in brain.

### Bacterial diseases

Bacterial infection was considered the main diagnosis in 11 submissions (13.9%) and 16 birds (13.3%). Avian pasteurellosis was found in two submissions involving two 6–7-months-old ducks. One owner reported 50% sudden mortality in domestic ducklings in one week. Typical gross and microscopic findings were observed at necropsy, including congestion, widespread septicaemic hemorrhages, fibrinous exudate in the coelomic cavity, moderate hepatosplenomegaly and necroses in liver, spleen and kidneys. *Pasteurella multocida* subsp. *multocida* was isolated from spleen. The other case was a Muscovy duckling which had shown signs of depression, tachypnoea and respiratory rales prior to death. Gross lesions were congestion and moderate hepatosplenomegaly and *Pasteurella multocida* subsp. *septica* was isolated from spleen. Another Muscovy duckling from a separate submission was diagnosed with *Riemerella anatipestifer*-infection. Weakness, eye infection and high mortality were mentioned at submission and fibrinous polyserositis was observed at necropsy. In two other submissions with 1–3-weeks-old ducklings (5 and 2 birds submitted, respectively) from flocks with high mortality, gross findings included congestion and widespread hepatocellular necroses. Colibacillosis was diagnosed in one case, while culture revealed growth of *Hafnia alvei* from one bird and mixed bacterial growth from another in the other submission. Additionally, pure moderate growth of *Aeromonas* sp. was found at culture from a Muscovy duck with hepatomegaly, cholangiohepatitis and splenitis.

**Fig. 3:**
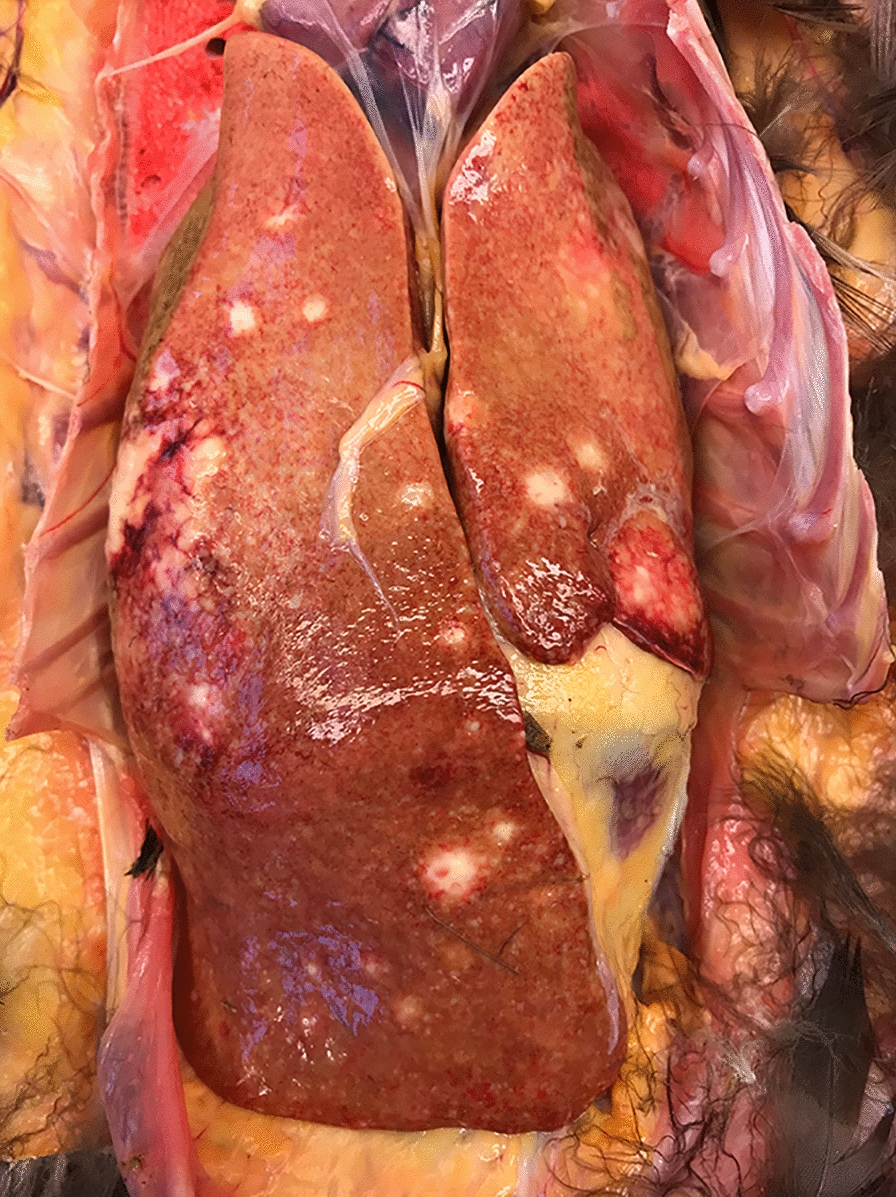
Pale and enlarged liver with multifocal coalescing nodules of different sizes in a domestic duck with avian mycobacteriosis

Mycobacteriosis (*Mycobacterium* sp.) was diagnosed in three submissions involving one domestic duck (Fig. 3) and two geese from two separate flocks. The birds were 2–5 years old and were in poor body condition or emaciated. Case histories and gross and microscopic lesions were suggestive of mycobacteriosis and the diagnosis was confirmed with Ziehl–Neelsen staining. Two other submissions with one bird each presented with granulomatous inflammation caused in the first bird by *Staphylococcus aureus* (granulomatous hepatitis, splenitis, pancreatitis, myocarditis and encephalitis with large numbers of intralesional cocci) and in the second bird by *E*. *coli* and *Enterococcus faecalis* (granulomatous pneumonia and airsacculitis).

### Fungal diseases

**Fig. 4:**
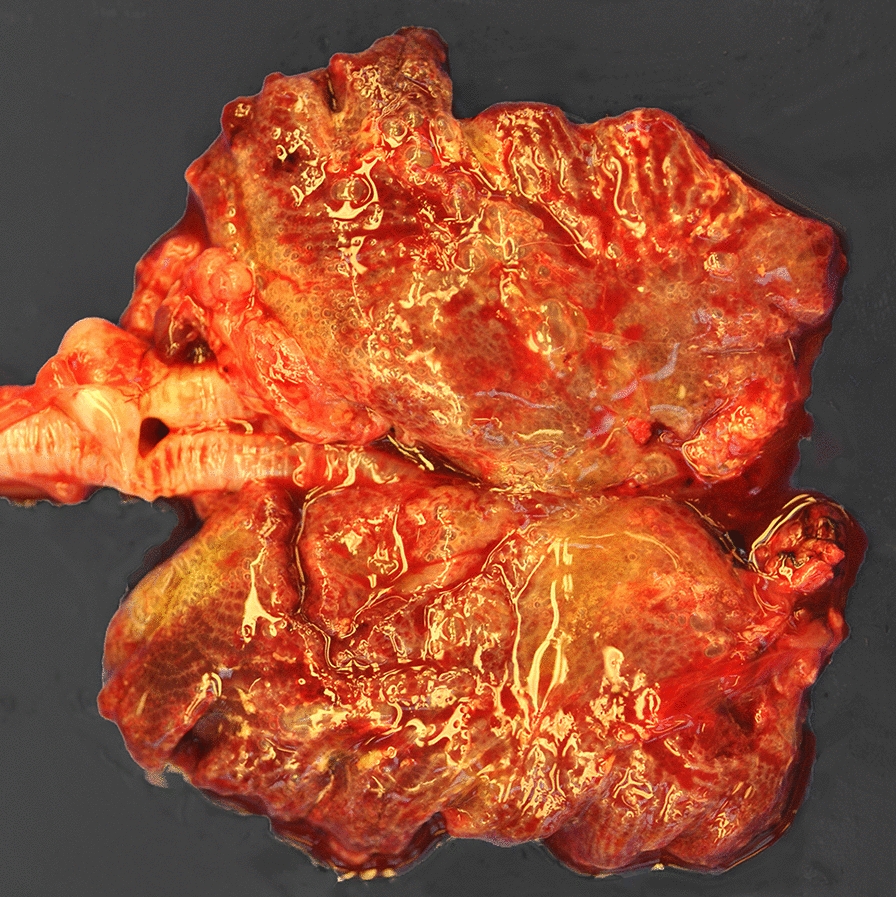
Severe pulmonary congestion in disseminated yeast infection in a Muscovy duckling

**Fig. 5:**
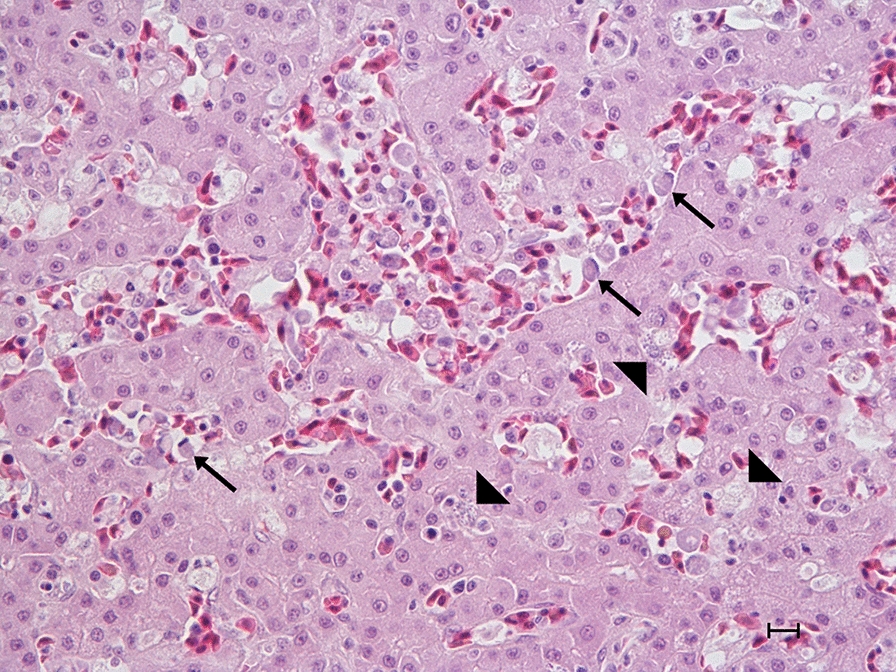
Concurrent leucocytozoonosis (parasites in blood cells, arrows) and disseminated yeast infection (intraendothelial yeasts, arrowheads) in the liver of a domestic duck/Muscovy duck hybrid duckling. HE staining, bar = 10 μm

Fungal infection was diagnosed in eight submissions (10.1%) and 12 birds (10.0%). Disseminated yeast infection i.e., Muscovy duck disease, was diagnosed in four submissions involving a total of five Muscovy ducks and one hybrid duck. Affected birds were 4–10-weeks-old and arrived in July–November from three counties in southern Sweden. Owners reported sudden onset of weakness, inappetence, laboured breathing, watery/green diarrhoea, and high flock mortality (35–100%) in ducklings while adults remained unaffected. At necropsy, five out of six birds were thin, whereas the remaining bird was in normal condition. The lungs were dark pink, severely congested and numerous air bubbles were observed on the surface (Fig. 4). Right-sided cardiac dilatation was present in several birds. The spleen and liver were enlarged, and the gall bladder was distended. Fibrin strands were present on thoracic airsacs, and clear coelomic and pericardial effusion, epicardial and splenic haemorrhages and intraureteral urate deposits were observed in some birds. Microscopy revealed 1–2 µm large round to oval Gram-negative, PAS- and Grocott’s methenamine silver-positive organisms in large numbers intraendothelially in capillaries in the parabronchial wall, and in lesser numbers in other tissues including liver (Fig. 5), spleen, intestine, myocardium, kidney, trachea and brain. There were marked atrial and parabronchial proteinaceous oedema in some birds and interparabronchial septae were distinctly widened from accumulation of fluid. There were no signs of necroses. A sparse inflammatory infiltrate consisting of lymphocytes and plasma cells was present in interparabronchial septae and parabronchial walls in occasional birds. Aerobic cultures from two submissions (two birds) revealed growth of *E. coli* or *Aeromonas hydrophila* from lung or spleen, respectively, but routine cultures for yeasts (lung tissue from two birds) were negative. PCR assays on lung, spleen and liver samples from three birds/two submissions were positive with both primer combinations. Sequencing of the partial 18S rDNA (579 bp) PCR product from lung from one of the Muscovy ducks in this study (GenBank accession number gb|MW675845) showed 98.1 and 99.1% sequence similarity to the yeast sequences (gb|FJ848337.1 and gb|KM103295.1) from a great blue heron (*Ardea herodias*) and a Muscovy duck from Canada [18, 19], respectively. PCR assay targeting *Leucocytozoon*, *Plasmodium*, and *Haemoproteus* was negative.

Aspergillosis was diagnosed in a subadult domestic duck with granulomatous pneumonia, caseonecrotic plaques on airsacs and mesentery and sporulating fungal growth in thoracic airsacs. The bird had shown signs of weakness, loss of body condition and anorexia, and there was 20% flock mortality. A second case was diagnosed in an adult goose with loss of body condition, weakness and inappetence. At necropsy, airsacculitis, pericarditis, epicarditis and hydropericardium were observed and fungal growth was detected intratracheally with microscopic features consistent with aspergillosis.

Characteristic microscopic features of pulmonary invasive zygomycosis were observed in a submission involving two adult Muscovy ducks in fair body condition and with regressing ovary. Gross findings included white discoloration of lungs, thickened airsacs, serosanguinous coelomic fluid, pale swollen liver and kidneys and multiple pale yellow 2 mm large nodules on the intestinal serosa and mesentery. Shell gland impaction/salpingitis was noted in one bird. In addition to zygomycosis, microscopy revealed systemic amyloidosis, coelomitis, granulomatous enteritis and renal tubular necroses. The causative fungus was not identified as cultures were not ordered. Further, profuse invasive fungal growth was identified in the trachea in a two-months-old domestic duck with leucocytozoonosis. The fungal species was not identified as no cultures were made. Yeast infection in oesophagus and crop was diagnosed at microscopy in a four-week-old gosling in below normal body condition.

### Parasitic diseases

In total, parasite infection was the main diagnosis in 12 submissions (15.2%) and 23 birds (19.2%), of which one submission with four goslings was diagnosed with two unrelated parasite infections considered to contribute to mortality (see below). Leucocytozoonosis was diagnosed in nine submissions (11.4%) involving 17 birds (14.2%). Cases were found among domestic ducks (two submissions, two birds), Muscovy ducks (five submissions, 13 birds), a hybrid duck (one submission, one bird) and geese (one submission, one bird). Leucocytozoonosis was confirmed by PCR (lung, liver and spleen, pooled as per submission and tissue) in three randomly selected submissions involving nine Muscovy ducks. Cultures for bacterial pathogens revealed no or nonspecific growth. Concurrent findings were tracheal mycosis in one domestic duck and disseminated yeast infection in the hybrid duck (Fig. 5). Cases arrived in June–August in five separate years from six different counties in southern and central Sweden. Affected birds were ten days to two months old and mortality in this age group ranged from 20–100% within a few days to a few weeks prior to submission, while adult birds remained unaffected. Most owners described that whole broods died within a few days. Birds exhibited signs of weakness, ataxia, inappetence, green diarrhoea, and laboured breathing, and death followed within a few hours. In some cases, the birds were found dead without prior signs of disease. Access to outdoor facilities and ponds or streams were mentioned by most owners. At post-mortem examination, birds were thin and anaemic and gastrointestinal contents were scarce and often bilestained. Carcasses appeared oedematous, and there was clear to serosanguinous coelomic fluid in small to moderate amounts. Lungs were oedematous and pink to red and moderate to severe hepatosplenomegaly was observed. Spleen and liver were dark red and friable and focal white spots were occasionally present. Pericardial effusion and cardiac dilatation (predominantly left-sided) were regularly observed. Microscopically, and depending on the stage of the parasite life cycle at death, intraparenchymal megaloschizonts and/or intracellular gametocytes were observed. Megaloschizonts were present in various tissues such as liver, spleen, kidney, lung, myocardium, muscularis externa of the gastrointestinal tract, skeletal muscle and brain. Gametocytes were identified as intraerythrocytic or intraleukocytic foreign structures. Haemosiderosis and erythrophagocytosis were observed in spleen and liver in some cases.

Amidostomiasis (*Amidostomum anseris*) was diagnosed as contributing to mortality in two submissions involving a total of five goslings. There was increased mortality in both flocks. The four birds in one of the submissions were heavily coinfected with renal coccidia (*Eimeria truncata*). Small intestinal coccidiosis (species not identified) was diagnosed in one submission in a domestic duckling from a flock experiencing high mortality in 5–6-weeks-old ducklings.

Incidental parasite findings included *A*. *anseris* in three submissions involving two geese and three domestic ducks, tape worms and leaches in one common duck each (different submissions), and lice and mite infestation in two and one submissions in geese, respectively. Information on parasite status was missing in eight submissions (10.1%).

### Nutritional and management-related diseases

Nutritional and management-related diseases accounted for morbidity/mortality in 15 submissions (19.0%) and 17 birds (14.2%). There were seven submissions (8.9%) and seven birds (5.8%, domestic duck *n* = 2 Muscovy ducks *n* = 2, domestic goose *n* = 3) with gross evidence of primary gastrointestinal obstruction or impaction associated with foreign objects made of plastics or metal, fibrous plant materials (grass or straw), wood shavings, sand/gravel or feed, or combinations thereof. The affected gastrointestinal segment varied between birds, from crop to colon, and there was dilatation of proximal segments. Two were adult birds, and the remaining were subadults.

Rickets was diagnosed in three submissions and five birds involving two ducklings and three goslings with reported flock mortality. There was no information on type of feed provided. Hepatic lipidosis was diagnosed in three submissions involving one domestic duck, one Muscovy duck and one domestic goose. The immediate cause of death in the Muscovy duck was liver rupture and internal haemorrhage. The goose was obese and presented with multifocal liver haemorrhages and end-stage kidney disease (see below). Emaciation of unknown cause was diagnosed in one goose with concurrent amidostomiasis and in one Muscovy duck.

### Generalized noninfectious diseases

Generalised noninfectious disease processes were diagnosed as the cause of mortality in 23 submissions (29.1%) involving 33 birds (27.5%). Renal/visceral and or articular gout was the predominant pathology in nine domestic ducks, three Muscovy ducks and two domestic geese, and cases occurred among all age categories. Incidental findings included non-specific hepatitis and enteritis in a subset of the birds. Renal and visceral urate deposits were observed in several additional birds but considered as secondary to other disease processes and not included in this section. End-stage kidney disease (renal sclerosis) was diagnosed in an 8-year-old obese goose with concurrent hepatic lipidosis (see previous section).

Idiopathic cardiac dilatation and congestive heart failure was diagnosed in one two-months-old domestic duck, two Muscovy ducklings and one adult domestic goose. Depression and respiratory distress were noticed by owners prior to death or euthanasia. There was one case of primary reproductive noninfectious disease in an adult female obese goose with regressed ovary. Degenerated egg materials (yolk, albumen, membranes and shell remnants) were present in the coelomic cavity with fibrous adhesions between the egg mass and organs, loops of small intestine and mesentery (internal layer and chronic coelomitis). Cloacal prolapse of unknown aetiology in combination with skin necrosis in the vent region and severe secondary colonic and cloacal dilatation were diagnosed in an adult female domestic duck. Severe amyloidosis was diagnosed in two Muscovy ducks with zygomycosis (see above).

Various idiopathic inflammatory conditions that could not be assigned a definitive aetiology due to lack of ancillary diagnostics or no identified cause were assigned to the generalised noninfectious category akin to a previous paper [6]. Cases in geese included interstitial nephritis (*n* = 2), conjunctivitis/epicarditis/pneumonia (*n* = 2), necrotising typhlitis/coelomitis (*n* = 1), and granulomatous splenitis (*n* = 1). In Muscovy ducks there were two birds with oesophagitis, one with non-heterophilic perivascular encephalitis and one with haemorrhagic enteritis.

### Neoplasia

Neoplasia was identified in two submissions (2.5%) involving two birds (1.7%). At necropsy of an adult female Muscovy duck/domestic duck hybrid in poor body condition, a 13 cm large coelomic tumour consisting of fibrous stroma admixed with numerous cysts and extensive haemorrhage and necrosis was found in cranioventral position to the kidneys. Blood was present in the body cavity from the ruptured tumour. A definitive microscopic diagnosis was not achieved, but gross appearance and cell morphology were suggestive of a sex chord stromal tumour i.e., a granulosa cell tumour. The other case was a locally invasive scirrhous adenocarcinoma, which accounted for the death of a 3-year-old domestic duck which died from secondary small intestinal obstruction. A similar but smaller tumour was observed in caecum.

### Congenital/developmental diseases

**Fig. 6:**
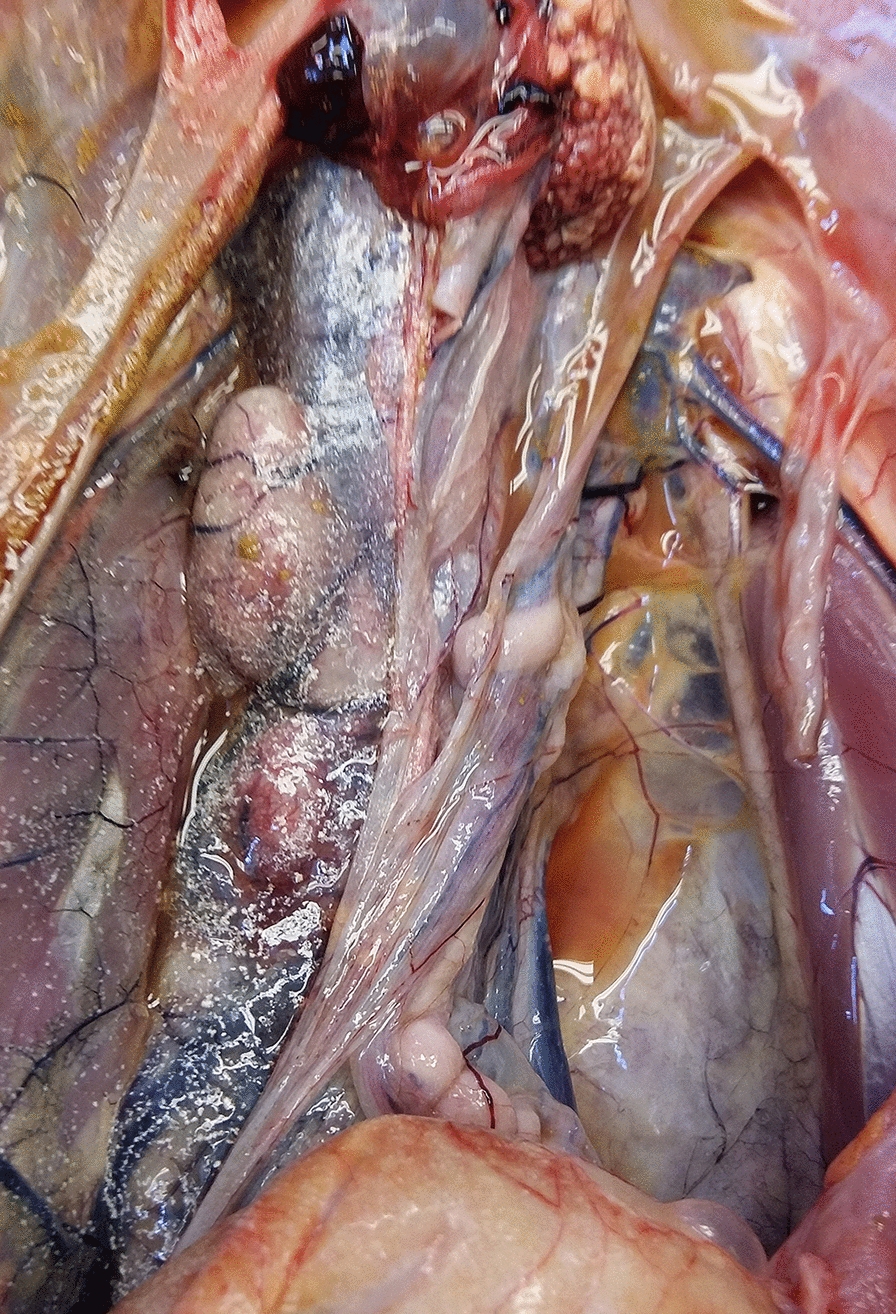
Unilateral renal agenesis (left kidney) with contralateral renal hypertrophy and visceral gout (urates visible on airsacs) in a domestic goose

Congenital/developmental anomalies were diagnosed in four submissions (5.1%) and four birds (3.3%). Ventricular septum defect (VSD) with signs of congestive heart failure was diagnosed in a 7-weeks-old gosling. Another diagnosis in this category was unilateral renal agenesis with compensatory hypertrophy of the contralateral kidney in a two-year-old goose (Fig. 6), which died from kidney failure as demonstrated by profuse visceral urate deposition (visceral gout). Leg-deformity was observed in one subadult duck with shortened tarsometatarsal bones and malformation of tarsal joints, and bilateral varus deformity was observed in a subadult domestic duck.

### Toxic diseases

**Fig. 7:**
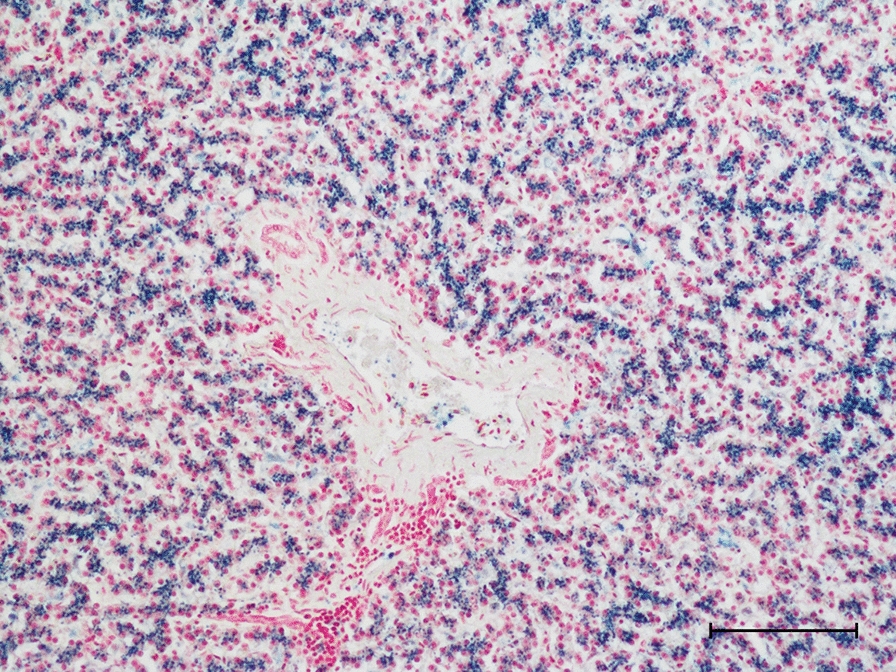
Marked hepatic haemosiderosis in a suspected case of garlic poisoning of a domestic duck. Pearl’s iron stain, bar = 100 μm

Two adult domestic ducks were submitted from a flock with increased mortality (three out of seven birds had died within a week before submission), egg drop, diarrhoea, listlessness, and ataxia. No gross pathological findings were recorded at necropsy. In one of the birds, microscopic examination revealed extensive hepatic haemosiderosis and degenerative lesions/necrosis in skeletal muscle in one of the birds. Kupffer cells and hepatocytes stained strongly with Pearl’s iron stain (Fig. 7) suggesting excessive iron deposits caused by nutritional iron overload or haemolytic anaemia. The other bird was examined in a later disease stage, and iron deposits in liver were present but less pronounced. A few days prior to the first symptoms, the birds had gained access to a vegetable plot and consumed large amounts of garlic and based on a previous case in white Chinese geese that were fed green onions [20], garlic-induced haemolytic anaemia was suspected.

### Trauma

Trauma was diagnosed in two submissions (2.5%) involving two birds (1.7%). One adult domestic duck died from predation. Based on lesion characteristics, a mustelid mammal was the most likely predator. The bird may have been weakened from concurrent chronic hepatis and amyloidosis, which were also observed at post-mortem examination, but these were not severe enough to be considered as contributing to mortality and there was no clinical history suggesting underlying disease. The other bird was a Muscovy duck with a large haematoma on the head and along the neck.

### Undetermined

In four submissions (5.1%) and five birds (4.2%) the pathologist was unable to determine the cause of mortality. These included three domestic ducks and two Muscovy ducks.

## Discussion

In this paper we have retrospectively reviewed consecutive post-mortem reports of 120 domestic ducks, Muscovy ducks, hybrid ducks and domestic geese from hobby/non-commercial poultry flocks submitted for routine diagnostics to SVA during a 10-year period (2011–2020). This study is one of very few published post-mortem case series from non-commercial poultry and only a subset of these earlier studies have included anseriform birds [6, 21, 22]. None of the previously published studies have focused solely on ducks and geese. Moreover, it is the first report of its kind from Scandinavia except a paper from Finland involving backyard chickens [5].

The number and geographic distribution of domestic anseriform birds is not known in Sweden. For this reason, conclusions regarding disease occurrence and regional representation of the population must be drawn with some care. Non-commercial poultry ownership is thought to have grown considerably in North America and Europe in recent years [2, 4, 5] and despite lack of population figures, this is most likely also true for Sweden. It is unknown whether this increase also involves anseriform birds, but it seems reasonable since they are often found in small numbers in mixed-species poultry flocks [23]. Resources therefore need to be allocated to ensure animal health and welfare not only on commercial farms but also in hobby poultry. Another motive is that small flocks may act as reservoirs of pathogenic and opportunistic microorganisms and transmission to commercial flocks is a possibility. Analysing necropsy results is one way to gain insight into disease occurrence in small poultry flocks. This paper presents diseases and other problems on small farms with domestic anseriform poultry and provides information that may help to improve flock health care. It is, however, important to remember that birds submitted for laboratory post-mortem examination often represent mortality events rather than day-to-day mortality. In this study, more than 72% of submissions involved flock mortality events rather than single dead birds. A major constraint of studies relying on privately owned birds is that authorization to perform necessary diagnostic tests may not be granted by owners for financial reasons. In this study this was often the case, despite that financial support was available for co-funding post-mortem examinations and some ancillary diagnostic tests in case of mortality at flock level. This problem, together with absence of routine surveillance activities in non-commercial poultry e.g., for *Salmonella* spp. and *Chlamydia* spp., may lead to underestimation of diseases and infections.

In general terms, husbandry methods in non-commercial poultry including ducks and geese differ considerably from those applied by the poultry industry [23]. Factors that may contribute to a different disease profile among small non-commercial flocks include for example unregulated trading of eggs and birds, mixing of birds of different species, ages and origins, presence of subclinical carriers and missing or inadequate biosecurity routines. It is also likely that ducks and geese in small non-commercial flocks have access to the outdoor environment with ponds or natural waters such as streams, lakes or coastal habitats, and thus, contact with wild free-living waterfowl may be exceedingly difficult to avoid.

The overall disease profiles seemed to differ between domestic ducks, Muscovy ducks and geese in this study (Fig. 1). For example, bacterial diseases were diagnosed more often in domestic ducks than in Muscovy ducks and domestic geese, whereas parasitic diseases were more common in Muscovy duck (including hybrids) and geese compared to ducks. Also, fungal diseases were most common in Muscovy ducks. These differences may be partly explained by the low numbers of birds investigated in this study, but there may also be true differences in susceptibility to infectious agents and other diseases. One such example is systemic yeast infection, which mainly occurs in Muscovy ducks. Management-related factors may also influence disease occurrence. Domestic ducks and Muscovy ducks often have access to water sources such as ponds and streams from an early age where there is a risk for transmission of pathogens from the wild avifauna and arthropod vectors, while geese are more often raised on pasture without access to natural waters. Moreover, it is easier and less expensive to transport small ducks compared to adult heavy geese to the laboratory, which also may influence disease profiles.

Several diagnoses in this paper represent the first reported occurrence in anseriform poultry from Sweden. Some examples include DVE, HPAI subtype H5N8, systemic yeast infection, leucocytozoonosis and *Riemerella anatipestifer* infection. This may reflect the small anseriform population, the low numbers of anseriform poultry submitted for post-mortem diagnostics and lack of previous reports rather than recent disease introductions. Duck virus enteritis has been previously reported from a range of duck-producing countries including China, Vietnam, Bangladesh, India, USA, Canada, The Netherlands, the United Kingdom, France, Belgium, Germany, Poland, Denmark and Egypt [24]. The DVE cases in Muscovy ducks in our study were epidemiologically unrelated and birds displayed typical gross and microscopic findings except for absence of lesions in the oral cavity and oesophagus. No spread to other flocks was documented. The source of the virus most likely was wild free-living asymptomatic carrier waterfowl either by direct contact or through contaminated water in cohabited ponds. Although DVE has never been diagnosed in wild waterfowl in Sweden, the causative virus (DEV) has been detected at high rates in wild free-living waterfowl in Europe [25].

The HPAI H5 clade 2.3.4.4 were spread to Europe in 2014 onwards and has caused numerous outbreaks in wild free-living birds and poultry, including in Sweden [26]. One of these outbreaks occurred in a small non-commercial mixed flock and involved chickens and Muscovy ducks. Few cases have been previously reported from domestic ducks [27, 28], but together with our case they suggest that domestic ducks may be highly susceptible to this particular virus clade.

Leucocytozoonosis is caused by a haemosporidian parasite (presumably *L*. *simondi*) known to infect and cause disease in wild free-living as well as domestic anseriform birds in North America and Europe. The sporozoite stage is transmitted by haematophageous arthropod vectors of genus *Simuliidae* (blackflies) and they develop into gametocytes in erythrocytes and leucocytes and megaloschizonts in reticuloendothelial cells in a variety of organs and tissues, most often observed histologically in liver, spleen and myocardium. We observed that leucocytozoonosis was diagnosed in duckling and goslings in summer (June–August), which probably reflects seasonal parasite reactivation in adult wild or domestic carrier birds in combination with vector exposure and availability of susceptible young birds. A seasonal relapse phenomenon i.e., parasitaemia in adult birds appearing during the breeding season, has been shown to exist in avian haemosporidian parasites [29]. Gross findings in the affected young ducks and geese of this study reflected the effects of severe haemolytic anaemia, and mortality is often very high and may reoccur in the same flock in different breeding seasons unless young birds are protected from the vector.

Until recently, disseminated yeast infection, also known as Muscovy duck disease, was of unclear aetiology. Following a case report from Canada involving a great blue heron (*Ardea herodias*) and molecular identification of an intracellular yeast belonging to the *Saccharomycetales* family [18], a closely related yeast was reported from Muscovy ducks in Canada [19]. Presumed earlier cases have also been reported in Muscovy ducks and domestic ducks [e.g. 30, 31]. In this paper we report additional cases in Muscovy ducks and in a Muscovy duck/domestic duck hybrid, and a closely related yeast sequence from one bird. Further, a retrospective analysis of anseriform necropsies performed at SVA in 1992–2010 (unpublished) identified two additional outbreaks, both diagnosed in 1993 in Muscovy ducks, which had been incorrectly diagnosed as leukocytozoonosis. These early cases occurred in northern Sweden, including one outbreak in the farthermost northern county. Interestingly, gross findings of coelomic fluid accumulation and hepatosplenomegaly in disseminated yeast infection are reminiscent of leucocytozoonosis. These two diseases both occur in young anseriform birds in summer/autumn and may even concurrently affect the same bird, as demonstrated in this paper. Discriminating findings include severe lung pathology in disseminated yeast infection and anaemia in leucocytozoonosis and the respective organism may be provisionally identified microscopically. The epidemiology and pathogenesis of disseminated yeast infection in anseriform birds remain poorly understood. However, the predominant pathology, especially the profuse intraendothelial yeast invasion and severe pulmonary oedema in the absence of necrosis and marked inflammation, suggests that the infection leads to occlusion of parabronchial wall capillaries and severely impaired gas exchange and secondary cardiac dilatation with pulmonary and coelomic fluid accumulation.

Another rarely reported fungal infection in anseriform birds, i.e., zygomycosis, was diagnosed in this study. The affected Muscovy ducks presented with lesions comparable to descriptions in an earlier report from ducks [32], but with the additional finding of systemic amyloidosis.

Gastrointestinal impaction is a well-known problem in non-commercial poultry including anseriform birds [6, 33, 34]. This study lends further support to the need of protecting ducks and geese from access to foreign objects, long dry grass and expandable feed, to provide drinking water at all times and to avoid stress, which may induce birds to ingest excessive volumes of inappropriate materials. Another management-related disease is rickets, which was diagnosed in both ducks and geese in this study. The dietary needs of non-commercial poultry at different life stages, not least among fast-growing young anseriform birds, are not always appreciated by owners.

Deposition of urates in kidney parenchyma, ureters, on serosal surfaces and in joints i.e., gout, is the end result of renal malfunction and hyperuricaemia in birds. It may have a multitude of aetiologies such as dehydration, postrenal obstruction, excess dietary proteins or calcium, water deprivation and infections [6, 35], but at necropsy it is rarely possible to determine the cause in individual birds. In this study, gout was diagnosed in domestic ducks, Muscovy ducks and domestic geese and in different age categories, which supports earlier findings that it is a common cause of mortality in poultry, independent of species [6].

In agreement with earlier reports describing causes of mortality in non-commercial poultry, congenital and developmental anomalies were diagnosed at low frequency in this case series [6, 22]. Unilateral kidney agenesis is a rare, although well-known, incidental congenital anomaly in poultry, which is occasionally diagnosed in birds with compensatory contralateral renal hypertrophy and visceral urate deposits [6]. It is, however, not clear whether unilateral renal agenesis leads to nephron overload and renal failure in birds.

A distinct seasonal submission pattern was observed in this study, with 57.5% of the birds arriving for necropsy in June to August. This may be associated with our findings of infectious diseases in young anseriform birds, such as leucocytozoonosis and fungal infection that may cause flock mortality events in young birds and perhaps higher interest among owners to achieve diagnoses in young birds.

## Conclusions

A wide range of different causes of mortality was observed in this study, some of which likely were a result of transmission from wild free-living waterfowl or by arthropods, while others were associated with animal husbandry or idiopathic causes. Anseriform poultry are considered as minor poultry species and research on animal health and disease prevention is limited compared to gallinaceous species. This is particularly true for non-commercial birds. The information obtained in this study can be used to identify potentially important disease conditions and help owners and veterinarians prevent disease and provide adequate veterinary care for con-commercial anseriform poultry.

## Data Availability

The dataset used during the current study is available from the corresponding author on reasonable request.
